# Attitudes and values among the Swedish general public to using human embryonic stem cells for medical treatment

**DOI:** 10.1186/s12910-022-00878-6

**Published:** 2022-12-22

**Authors:** Åsa Grauman, Mats Hansson, Dag Nyholm, Elena Jiltsova, Håkan Widner, Trinette van Vliet, Jennifer Drevin

**Affiliations:** 1 Centre for Research Ethics and Bioethics, Uppsala University, Box 564, 751 22 Uppsala, Sweden; 2 Department of Medical Sciences, Neurology, Uppsala University Hospital, Uppsala University, 751 85 Uppsala, Sweden; 3 Department of Neurology, Skåne University Hospital, 221 85 Lund, Sweden

**Keywords:** Surplus embryos, Public perceptions, Parkinson’s disease, Drug development, Moral concerns

## Abstract

**Background:**

The use of human embryonic stem cells (ES cells) for the development of medical therapies is surrounded with moral concerns. The aim of this study was to assess the public’s attitudes toward the use of ES cells for treatment of Parkinson’s disease (PD) and other diseases, what factors are most important to consider when using ES cells for drug development, and if there is an association between religious beliefs and attitudes toward using ES cells for medical treatment.

**Methods:**

A randomly selected sample of the Swedish public, aged 18–87-years-old, completed an online survey (n = 467). The survey assessed socio-demographics, religious views, perceived moral status of the embryo, and attitudes toward using ES cells for medical treatment of PD and other diseases. Adjusted odds ratios (ORs) and 95% confidence intervals (CI) for positive vs. negative attitude toward using ES cells for drug development were computed using logistic regression.

**Results:**

The respondents were positive about using ES for treatment; specifically, 70% totally agreed that it is acceptable to use ES cells for treatment of PD, while 40% totally agreed that it is acceptable to use ES cells for treatment but induced pluripotent cells is just as efficient. Religion being of little importance in one’s life was associated with a positive attitude toward using ES cells for treatment of PD (adjusted OR 6.39, 95% CI 2.78–14.71). The importance of being able “to access new, effective treatments against diseases that do not have any treatment available” was ranked as the most important factor to consider when using ES cells for drug development.

**Conclusion:**

Most respondents are positive about using ES cells for drug development, and making effective treatments accessible to those who do not have any. However, these attitudes are influenced by the specific disorder that the drug development is intended for, as well as the religious views and perceived moral status of the early embryo.

**Supplementary Information:**

The online version contains supplementary material available at 10.1186/s12910-022-00878-6.

## Introduction

Stem cells are currently being used to develop therapies for a number of incurable diseases [1]. Human embryonic stem cells (ES cells) are immature, pluripotent cells that have the ability to divide an infinite number of times and develop into any specialized cell [2]. Generation of ES cells involves extracting cells from the blastocyst on the fifth day after conception. The embryos used to generate ES cells are often surplus embryos donated by couples undergoing in vitro fertilization (IVF) treatment. Couples undergoing IVF in Sweden can choose either to donate to other couples for reproductive purposes, to donate the embryos for research purposes, or to discard them. In Sweden, it is not allowed to use specifically created embryos for the purpose of research. Induced pluripotent cells (iPS cells) are created from reprogrammed specialized cells [3, 4]. Researchers hope that both ES cells and iPS cells can be used in medical treatment in the field of regenerative medicine to replace the cells that have been damaged or have died. Parkinson’s disease (PD) is one of the conditions, where the development of treatment has come the furthest [5]. Parkinson’s disease is a chronic progressive disorder that can decrease an individual’s independence and quality of life. The etiology of PD is still unknown, and there is currently no cure for PD. Instead, treatment aims to reduce symptoms and maintain a quality of life using medication and non-pharmacological interventions. Therefore, development of effective disease modifying treatments is important in order to help patients and their families. The potential and limitations of ES-derived and iPS-derived cell products in the treatment of PD is summarized in a recent review by Parmar et al. [1] There are currently three ongoing clinical trials, two with a cell product produced from ES cells and one with cells produced from iPS cells. A case report of one patient transplanted with an autologous iPS cell-derived product is also published [6].

Unlike, iPS cells, the use of ES cells is surrounded by certain moral concerns because early embryos are discarded during production. The view of the embryo’s moral status varies, depending on the outlook on life and has previously been the subject of extensive discussions [7–9]. Strong voices in society, from activists and religious leaders, have opposed the use of ES cells [10]. These moral objections have influenced legislation, including restricting the time for usage to 14 days in order to protect the special status of the unborn [11], and have in some countries led to banning research with ES cells [5]. Furthermore, manufacturing medical treatment involves commercialization, which risks embryos being considered as commodities, with a lack of respect for human life [12]. When the time comes for treatment with cell products derived from ES cells or iPS cells to be introduced on the market, policymakers or relevant qualified regulatory agencies need to decide whether commercialization of these cells should be allowed for treatment of patients, or under what conditions they should be allowed. Before such decisions are made, it is crucial that different publics’ views are considered, which is empathized in the Swedish Patent Act (1967:837, 2020:541) Chapter 1, Article 1c, laying down that "a patent is not granted for an invention where the commercial exploitation would be contrary to public order or morality".

Much of the previous research has focused on the IVF couples’ views. A systematic review from 2014 found that couples who donated their embryos to research perceived it as a better alternative than the destruction of embryos and as an opportunity to help others. Couples assigning embryos a high moral status were less likely to donate their embryos for research purposes [13]. In a Swedish cross-sectional study on the attitudes toward embryo donation, about half of the respondents were positive toward donating for research purposes [14]. In a Chinese study, 59% of the IVF couples preferred to discard surplus embryos rather than donate them to research, stating a lack of information and distrust in science as significant reasons for their decision [15]. A cross-sectional study assessing if Latin American IVF couples would donate embryos found that the attitudes varied depending on the specific purpose of donation. The respondents were most positive about donation for treatment with embryonic stem cells (73.6%), while 63.8% would donate to heterosexual couples for reproductive purposes, and 45.1% to single women [16], indicating that the purpose of donation matters for people’s attitudes. The source of the embryo, either being surplus of created for research purpose, also matters for peoples attitude for usage [17]. In a qualitative study from 2022 with members of the Swedish general population, the participants had a positive attitude toward using embryonic stem cells to treat PD; however, they raised several conditions under which the treatment should be allowed [18]. Some found early embryo donation to be completely unproblematic, while others had ethical concerns connected to their perception of the early embryo, in terms of being a “potential life.” The participants who viewed the early embryo as a potential life were still positive about donating, since they considered the benefit of helping PD patients find an efficient treatment as being more important than the negative aspects related to using the early embryos. However, some thought that ES cells would no longer be used if iPS cells or other treatments were as efficient. This emphasizes the importance of specifying the disease and the presence of available treatments when assessing the public’s attitudes toward using ES cells for treatment. In this study, we wanted to follow-up on the findings from the previous Swedish interview study to assess the prevalence of the attitudes and values in the Swedish population regarding the use of ES cells for drug development. The study’s aim was to study the general public attitude about the use of ES cells for medical treatment in general and of PD in particular, and what factors are most important to them when considering using ES cells for drug development. Further, we aimed to investigate if there is an association between the importance of religion in one’s life and attitudes toward using ES cells for medical treatment in general and of PD in particular.

## Methods

### Data collection and study population

This was a cross-sectional study among a random sample of Swedish citizens, aged 18 or older (n = 2755), drawn from the Swedish State Personal Address Register. The register includes Swedish residents. An invitation letter included information about the study, together with a link to an online survey. Thirty-four letters were not delivered. Data collection continued until we reached a minimum of 400 respondents. Of the total 2721 who received the invitation, 467 participated in the study (17%). Fourteen people informed us that they did not want to participate, five were not able to participate because of their health status, two did not have a computer, and one did not understand Swedish. Data were collected between March and May 2021.

### Research ethics

The study was approved from the Swedish Ethical Review Authority before the data collection started (Dnr 2019-06539). Informed consent was obtained from all the respondents. Data were presented in such way that no individual can be identified. All the methods were carried out in accordance with relevant national and international guidelines and regulations.

### The survey and variables

The online survey included questions about age, gender, country of birth (Sweden or other), occupation, highest level of education completed (later dichotomized as 1 = university, 0 = all other), pharmaceutical use, and health literacy (Sufficient or limited) [19].

After responding to these questions, the respondents were asked to read a text informing them about human embryonic stem cells and how they can be used to treat PD (Additional file 1).

Thereafter, the respondents were asked to rank eight statements regarding factors to consider when using surplus embryos for drug development based on importance (Table 4). The statements were formulated based on interests expressed by patients, the general public, and couples having cryopreserved embryos in previous qualitative interviews.

Attitudes toward using leftover embryos for drug development were assessed by three items and Likert scales ranging from 1 to 5 (1: fully agree, 2: highly agree, 3: partially agree, 4: highly disagree, and 5: fully disagree). The point of departure was a scenario where leftover early embryos from IVF treatment were donated by the couple for the specific purpose, without financial compensation. The statements the respondents were to decide upon were: It is acceptable that surplus embryos from fertility treatments are (1) used for treatment of Parkinson’s disease, (2) used for treatments of other types of diseases, and (3) used for treatment of diseases, even if induced pluripotent cells may be used with similar results. In the analyses, the ratings from the Likert scales were dichotomized into positive attitude (1–2 point) and negative attitude (3–5 point).

The respondents’ perception of the moral status of a couple of days old human embryo was assessed using the question, “The human is perceived to have a special moral position, in the sense of having rights just by being human. What moral position does a human embryo that is only a few days old have? Respondents could choose between four statements (The embryo is just a lump of cells = 1; it is meaningless to discuss its moral status / the embryo has a moral status that is in between being just a lump of cells and being a human being = 2; The embryo in its moral status is closer to being a human than just a lump of cells = 3); and the embryo has the same moral status as a human being = 4). The variable was dichotomized into low status (1–2 points) and high status (3–4 points).

The importance of religion in the respondents’ life was assessed on a 5-point Likert scale (1: very little importance, 2: fairly little importance, 3: neither great nor little importance, 4: quite great importance, and 5: great importance). The variable was dichotomized into little importance (1–3 point) and high importance (4–5 points).

### Statistical analyses

Descriptive statistics are presented with mean and standard deviation for continuous variables and as frequencies for categorical variables. The difference in attitudes toward using surplus embryos for drug treatment between the three purposes of use was tested with Wilcoxon Signed Ranks Test. The Mann–Whitney U test and Spearman’s correlation test were further used to test for differences in attitudes based on age, sex, education, country of birth, health literacy, moral status of the embryo, and religion. The associations between religion and attitudes toward using surplus embryos for drug treatment were estimated as odds ratios (ORs) with 95% confidence intervals (CI). Three separate binary logistic regression analyses were made, one for each attitude item. A negative attitude was the reference category. The crude model included religious views. Model 1 included variables in model 1 and age, sex, education, health literacy, country of birth, and use of pharmaceuticals. Model 2 included variables from the previous models, and moral status of the embryo. All analyses were performed using SPSS 25.

## Results

In this cross-sectional survey study, the age of the respondents ranged from 18 to 87 years and encompassed about an equal share of men and women. Almost half of the respondents had a university degree, and 86% were born in Sweden (Table 1).

**Table 1:** Characteristics of the respondents (n = 467)

	*n* (%)	Mean (SD)
*Age (years)*		49.9 (16.9)
18–30	75 (16.2)	
31–50	160 (34.6)	
51–65	125 (27.0)	
66–87	103 (22.2)	
*Sex*		
Male	235 (50.9)	
Female	227 (49.1)	
*Country of birth*		
Sweden	400 (86.4)	
Other	63 (13.6)	
*Education*		
No education	3 (0.6)	
Primary school	30 (6.5)	
Secondary school	135 (29.2)	
Vocational school	69 (14.9)	
University	226 (48.8)	
*Occupation*		
Working	287 (38.0)	
Retired	117 (25.3)	
Sick leave	7 (1.5)	
Parental leave	15 (3.2)	
Unemployed	21 (4.5)	
Student	44 (9.5)	
None	10 (2.2)	
*Pharmaceutical use*		
Daily	215 (46.4)	
1–6 times a week	31 (6.7)	
1–3 times a month	59 (12.7)	
< Once a month	124 (26.8)	
Never	34 (7.4)	
*Health literacy*		
Inadequate	15 (3.2)	
Problematic	143 (30.6)	
Sufficient	299 (64.0)	

About half of the respondents stated that religion had a very little meaning in their life, while only 4.4% stated that religion had very much meaning in their life. A majority of the respondents perceived the early embryo as a lump of cells, while 3.9% thought that the early embryo has the same moral status as a human being (Table 2).

**Table 2:** Descriptive statistics of meaning of religion and perceived moral status of the embryo (n = 467)

	*n* (%)
*Meaning of religion in your life*	
Very little	226 (52.1)
Pretty little	84 (19.4)
Neither little nor a lot	73 (16.8)
Pretty much	32 (7.4)
Very much	19 (4.4)
*Perceived moral status of embryo*	
The embryo is just a lump of cells; it's pointless to discuss its moral standing	252 (58.1)
The embryo has a moral position that lies in the middle of being just one lump of cells and being a human being	128 (29.5)
The moral position of an embryo is closer to being a human being than just a lump of cells	37 (8.5)
The embryo has the same moral status as a human being	17 (3.9)

### The public’s attitude toward using surplus embryos for development of medical treatment

The respondents were overall very positive toward using surplus embryos for drug development (Table 3; a low median and mean indicate a positive attitude). There was a statistically significant difference in attitude, based on the specific conditions of use. The respondents were most positive toward using surplus embryos for treating Parkinson’s disease specifically; second, most positive toward using surplus embryos; and least positive toward using surplus embryos, when iPS is just as efficient. The Mann–Whitney U test further showed that respondents who were less religious, male, younger, born in Sweden, and had sufficient health literacy were more positive toward using embryos. Respondents who perceived the embryo as a human or closer to a human than a lump of cells were most negative toward using embryos for drug development.

**Table 3:** Descriptive statistics of respondents’ (n = 435) attitudes toward using leftover embryos from IVF treatment for treatment, donated by the couple, without financial compensation for the purpose of drug development

Statement	Median (IQR)	1 Totally agree n (%)	2 Highly agree n (%)	3 Partially agree n (%)	4 Agree to a low degree n (%)	5 Do not agree at all n (%)
It is acceptable to use leftover embryos for treatment of Parkinson’s disease	1 (1)	306 (70.3)	89 (20.5)	20 (4.6)	6 (1.4)	14 (3.2)
It is acceptable to use leftover embryos for treatment of other types of diseases	1 (1)	284 (65.3)	100 (23.0)	25 (5.7)	13 (3.0)	13 (3.0)
It is acceptable to use leftover embryos for treatment of diseases but iPS is just as efficient	2 (2)	175 (40.2)	93 (21.4)	103 (23.7)	28 (6.4)	36 (8.4)

Descriptive statistics are presented using medians, inter-quartile ranges (IQR), and the distributions of responses n (%)

The associations of religious view and attitudes toward using surplus embryos were estimated in three separate logistic regression models for each attitude statement, shown in Table 4. Respondents who stated that religion had little importance in their life were more likely to have a positive attitude toward using surplus embryos compared to respondents with religion playing an important role in their lives. The association was strongest when surplus embryos were used for treating PD (adjusted OR 6.39, 95% CI 2.78–14.71), followed by treatment of other diseases (adjusted OR 3.47, 95% CI 1.56–7.71), and using embryos despite iPS cells being just as efficient (adjusted OR 1.75, 95% CI 0.92–3.34).

**Table 4:** The association of importance of religion in life with attitudes toward using embryos for treatment of I) Parkinson’s disease II) of other diseases III) of diseases, although iPS would be as efficient

	Crude model OR (95% CI)	Model 1 OR (95% CI)	Model 2 OR (95% CI)
*Positive attitude toward using embryos for treatment of Parkinson’s disease*			
Importance of religion in life			
Very little, fairly little, neither little nor a lot of importance	10.74 (5.21–22.18)	9.35 (4.34–20.12)	6.39 (2.78–14.71)
Fairly high, very high importance	1.00 (ref)	1.00 (ref)	1.00 (ref)
*Positive attitude toward using embryos for treatment of other diseases*			
Importance of religion in life			
Very little, fairly little, neither little nor a lot of importance	6.49 (3.31–12.73)	5.41 (2.68–10.94)	3.47 (1.56–7.71)
Fairly high, very high importance	1.00 (ref)	1.00 (ref)	1.00 (ref)
*Positive attitude toward using embryos for treatment of other diseases, although iPS cells are as efficient*			
Importance of religion in life			
Very little, fairly little, neither little or a lot importance	2.58 (1.42–4.69)	2.29 (1.24–4.23)	1.75 (0.92–3.34)
Fairly high, very high importance	1.00 (ref)	1.00 (ref)	1.00 (ref)

Associations are presented using odds ratio (OR) and 95% confidence intervals (CI), and with respondents finding religion to be of fairly high or very high importance, as the reference groups (ref)

*OR* odds ratio, *CI* confidence interval. The crude model included religious views. Model 1 included religious views, age, sex, education, health literacy, country of birth, and use of pharmaceuticals. Model 2 included religious views, age, sex, education, health literacy, country of birth, use of pharmaceuticals, and perception of moral status of embryo

The complete regression models are presented in the Additional file 2, indicating that respondents that perceive the embryo as a lump of cells or between a lump of cells and human were more likely to be positive toward using surplus embryos for treating PD (7.86 (3.43–18.02)). Neither age, sex, health literacy nor country of birth were associated with attitudes. Additional analysis found that respondents who stated that religion had little importance in their life were more likely to perceive the moral status of the embryo as lower (adjusted OR 4.61, 95% CI 2.27–9.36), Additional file 2: Table S5.

### Most important factors to consider when using ES cells for drug development

The respondents were asked to rank statements of aspects to consider when using surplus embryos for drug development based on importance (Table 5). To have access to new, effective treatments against diseases that do not have any, was considered the superior most important thing to consider. This was followed by the need to reduce the risk of severe side-effects, and the need for transparency from medical companies involved in the drug development.

**Table 5:** Statements reflecting what the public thinks is important to consider when using surplus embryos for drug development

Statements	Median (IQR)	Ranked as most important (n)
It is important to access new, effective treatments against diseases that do not have any	1 (2)	255
It is important to try to reduce the risks for severe side-effects associated with medical treatments	2 (2)	23
It is important with transparency and scrutiny if medical companies are involved in developing treatment with embryonic stem cells	4 (2)	32
It is important that legislators consider the different values in society when deciding on the use of embryos for medical treatment	5 (3)	45
Patients should be informed on how their treatment has been developed before deciding whether they accept treatment or not	5 (4)	17
If we allow embryos to be used for medical treatment, there is a risk that we, in the future, will allow unwanted usage	6 (3)	14
For moral reasons, it is preferable to use induced pluripotent stem cells instead of embryonic stem cells	6 (3)	25
Couples donating embryos should not be compensated economically for their donation	6 (4)	25

Respondents were asked to rank the statements from most important (1) to least important (8). Descriptive statistics are presented using medians, inter-quartile ranges (IQR), and the number of times the statement was ranked as the most important (n)

## Discussion

The respondents who answered the survey were generally very positive about using ES cells for treatment, especially when it comes to treatment of PD. They were a bit less positive concerning the treatment of other diseases. However, if iPS cells are available with equal efficacy, the respondents were less positive about using ES cells. This corresponds to the findings reported in our previous interview study with individuals from the Swedish population [18]. It is possible that individuals with a special interest in PD were more likely to respond to the survey, thus resulting in a bias in the sample. That could explain them being more positive toward accepting treatment for PD in particular. Another explanation is that the attitudes were biased by the information about PD at the beginning of the survey, making the respondents feel more strongly about PD. However, finding an effective treatment for diseases that lack an available treatment was ranked the most important aspect to consider when utilizing ES cells for treatment purposes, dominating all other factors considered. This can explain why treatment for PD was ranked higher than treatment for other diseases, since current PD therapies only address symptoms. The results were not surprising as it corresponds to a previous qualitative study, where the respondents did not distinguish between PD and other serious chronic diseases lacking a curable treatment [18]. However, the respondents had a low ranking for the importance of using iPS cells instead of ES cells to produce medical products for moral reasons, indicating that, although it was considered important, other aspects are more important. However, there were 25 respondents (5.7%) who ranked this as the most important aspect; these respondents were probably more religious and perceived the moral status of the early embryo as a human or close to a human.

In this study, we found an association between attitude toward using ES cells for medical treatment purposes and religious views and the perceived moral status of the early embryo among the Swedish general population. A Swiss study found that IVF couples’ perception of the moral status of an embryo and their stated religious views were independently predictive of their attitudes toward donation of surplus embryos for various purposes [20]. The number of people in our study who stated that religion was important or very important in their life, and the number of respondents who viewed the moral status of the embryo as a human or close to a human, were relatively few (11.4% and 12.6%, respectively). According to the Culture Map, provided by the World Value Survey, Sweden is a country with the most secular values in the world [21]. The culture map also shows that the values of migrants from e.g., Afghanistan, Iraq, Somalia, living in Sweden differ substantially compared with native Swedes [21]. Sharma et al. found that Asian immigrants in the United States were less likely to donate surplus embryos for research use [22]. In our study, about 14% of the respondents were born outside of Sweden, but we do not know from which countries.

The assessment of religious concerns in this study may be seen as a limitation, and, indeed more nuanced views may have been revealed if we had used an instrument and questions from life view research that captured religious beliefs, morally salient values, as well as fundamental attitudes as a basic component of a religious worldview [23, 24]. However, the aim was to see if the respondents self-reported as seeing religion as something important in their lives, whether their views were influenced by beliefs about the world and human beings, their morally salient values, or basic attitudes related to if they had a more positive or a more negative view of life. The instrument used did, in fact, reflect both on beliefs and values, e.g., the biological and moral status of a human embryo. Furthermore, previous studies have assessed religious views in similar ways, also finding that individuals with moderate to strong religious beliefs were less likely to donate to research [25]. Further empirical explorations in life- and worldview research may provide a richer understanding of how both comprehensive and fragmented religious views may direct moral positions. It may also be noted that from other religious contexts, the views are both aligned with and different from the majority view in this study. For instance, Sivaraman and Noor have studied ethical views about using ES cell among religious leaders from different religions in Malaysia. They found that the Islamic and Hindu leaders allowed the use of surplus embryos, to which they attached less moral weight, while they prohibited the use of “research embryos.” Furthermore, Islamic leaders have concluded that the use of ES cells is allowed for the purpose of maintaining health, and mandatory if it can relieve people’s suffering. The Buddhist leaders in their study did not see any ethical difference between the use of either source of embryos, approving the use of ES cells as long as it is done with caution and care. The Catholic leaders, however, opposed the use of embryos, whether surplus or research embryos. The different standpoints reflected different views on the moral value of the embryo, and whether the benefit is more important than the harm. They also found different reflections from leaders from the same religion, expect the Catholic leaders, who repeated the same message as formulated by the Vatican [17].

Besides religious views and the moral status of the early embryo, other studies have found that men, in general, are positive about allowing embryo donation for research [14, 26]. Likewise, the univariate analyses in this study showed that men held a more positive attitude. However, this association was not found in the adjusted analyses. Furthermore, health professionals have been more positive than the public in previous studies on donating embryos for research [26]. Being scientifically oriented and having trust in experts and the medical system are also factors related to a more positive attitude [13, 18]. A limitation of the study is that we have not assessed the respondents’ trust in the healthcare systems or whether the respondents worked within the healthcare systems themselves. Neither does the questionnaire assess the importance of considering genetic privacy issues for donors or issues regarding commercialization. Another limitation is the low response rate (17%), restricting the generalization of the results to the Swedish population at large. Our study sample consisted of more individuals with a university degree compared with the Swedish general population. However, a random sample of the general population was invited to participate, and the final sample included an equal number of men and women of various ages and educational- and occupational backgrounds, which is a strength of the study. Therefore, although migrant groups may be missing, it is possible to make a statement about the attitudes and values of native Swedes of various ages.

The literature on the ethics of using stem cells give witness to moral concerns and sometimes to intense discussions [27, 28]. Some areas where this is evident are gene therapy, pre-implantation genetic diagnosis, whole genome sequencing, or gene editing. They have all, like stem cell-based research, stirred intense ethical discussions when they first were presented in scholarly journals and reported in public media. Some early research applications with these technologies were indeed premature and should have awaited better evidence but, after some progress and more scientific evidence about benefits and risks, most of them will belong to mainstream medical science. Gene therapy is an example of a promising new technology developed 40 years ago. It met quite some resistance, not the least from religious representatives. Some warned against Gene therapy as a way of “Playing God” [29]. Forty years later, there are approved treatments and several clinical trials with gene therapy ongoing. The technology is now moving into the mainstream medical science governed by ordinary regulatory frameworks for clinical trials, despite the fact that in the beginning it was viewed by many as being morally unacceptable. It is against this background that the results of this study are of particular interest, showing that the majority of the general public care foremost about issues relating to medical needs and being able to improve medical treatment. Furthermore, the history of stem cell research have shown us that strong moral values och opinions from some voices in society, e.g., activists or religious leaders, can influence legislation and stop development of valuable treatments for patients. For democratic reasons, it is crucial to make an effort to listen to the voice of the general public as direct stakeholders, and not only those with openly voiced opinions. It is not apparent that policy should always do as the majority thinks, but policy makers should always listen to their perspective, as part of a transparent dialogue to maintain trust for researchers, the health care system and the pharmaceutical industry. It is our hope that policy makers use the findings from this study for deliberation and communication.

## Conclusion

The majority of the respondents are positive towards using ES cells for drug development, and value making effective treatments accessible for those who do not have any. However, these attitudes are influenced by what disorder the drug development is intended for, and religious views and the perceived moral status of the early embryo.

## Supplementary Information

**Additional file 1.** Information provided to respondents after background questions.

**Additional file 2.** Additional statistical analyses.

## Data Availability

The datasets generated and/or analyzed during the current study are not publicly available since they contains sensitive information, but are available from the corresponding author on reasonable request.
