# Supplementary material for: Attitudes and values among the Swedish general public to using human embryonic stem cells for medical treatment
Source: BMC Med Ethics. 2022 Dec 22;23:138. doi: 10.1186/s12910-022-00878-6 (PMC9773498; doi:10.1186/s12910-022-00878-6)
Supplement: Supplementary file 1 — Additional file 1. Information provided to respondents after background questions. [file 12910_2022_878_MOESM1_ESM.docx]

**Additional file 1. Information provided to respondents after background questions. Originally provided to respondents in Swedish.**

## Part 2. Information on how embryos can be used to treat diseases

Below you will find a short informational text. The text provide you with a background for the last questions we will ask you.

**Information on how embryos can be used to treat diseases**

**Stem cells**

When a cell dies, it needs to be replaced. For that stem cells are needed. Stem cells can be specialized to varying degrees. There are, for example specialized stem cells that create new blood cells. Other types of specialized stem cells are found in the brain and skin.

**Embryonic stem cells (ES cells)**

There are also stem cells that are not specialized, so-called embryonic stem cells (ES cells). They can split any number of times and can, if they are grown and controlled in their development, replacing basically any cell in the body. With the help of ES cells, researchers are working to develop cell-based treatments to treat diseases such as Parkinson's disease.

**The use of embryonic stem cells to develop medical treatment**

To produce ES cells for development of treatments, cells from embryos are needed. An embryo is an egg that has been fertilized by a sperm. The embryos used has been left over and donated by couples who have undergone in vitro fertilization (sometimes called IVF or in vitro fertilization), to get pregnant. When the cells are taken from the embryo, the embryo is destroyed. The development of the ES cells can then be controlled, and the researchers can grow the kind of cells needed to develop a medical treatment. An embryo can give rise to a cell line that can be used to develop treatment for different types of diseases, and for the treatment of many patients.

**People with Parkinson's disease may benefit from the treatment**

Parkinson's disease is caused by a lack of a signaling substance called dopamine. The lack of dopamine leads to, among other things, involuntary tremors, muscle stiffness, difficulties in starting movements and that the movements take place more slowly. Other symptoms are reduced sexual desire, constipation, urinary incontinence and depression. There is currently no cure for Parkinson's disease but there are treatments that can reduce symptoms. By using ES cells to make cells that produce dopamine, researchers hope avoid future deterioration and repair damage caused to people with Parkinson's disease.

**Other cells than embryonic stem cells can also be used**

Other cells can also be used to grow cells that produce dopamine. By taking specialized cells, such as skin cells, and modify them, scientists can cause them to reverse in development and become unspecialized again. Cells that have been modified in this way are called induced pluripotent stem cells, or iPS cells. iPS cells can, just like embryonic stem cells, divide any number of times and are believed to be able to develop into basically any kind of cell. Researchers hope that also dopamine-producing cells developed from iPS cells should be able to be used for to treat people with Parkinson's disease.

**Pharmaceutical companies may make money from treatments with cells**

Pharmaceutical companies work to produce and develop new treatments. A safe and effective treatment requires a long and costly development time. In order to pay for the development of medicines, pharmaceutical companies need to be able to earn money on their products, even those based on donated skin cells and human embryos.
