# Supplementary material for: Attitudes and values among the Swedish general public to using human embryonic stem cells for medical treatment
Source: BMC Med Ethics. 2022 Dec 22;23:138. doi: 10.1186/s12910-022-00878-6 (PMC9773498; doi:10.1186/s12910-022-00878-6)
Supplement: Supplementary file 2 — Additional file 2. Additional statistical analyses. [file 12910_2022_878_MOESM2_ESM.docx]

**Additional file 2**

Table S1. The association of importance of religion in life with a positive attitude toward using embryos for treatment of I) Parkinson’s disease. Associations are presented using odds ratio (OR) and 95% confidence intervals (CI), and with respondents finding religion to be of fairly high or very high importance, as the reference groups (ref).

|  | Crude model  OR (95% CI) | Model 1  OR (95% CI) | Model 2  OR (95% CI) |
| --- | --- | --- | --- |
| Importance of religion in life (very little, fairly little, neither little nor a lot) | 10.74 (5.21- 22.18) | 9.35 (4.34- 20.12) | 6.39 (2.78-14.71) |
| Age |  | 0.98 (0.961-1.01) | 0.99 (0.97-1.02) |
| Sex (male) |  | 1.64 (0.77-3.46) | 1.68 (0.76-3.71) |
| Education (university) |  | 1.38 (0.65-2.93) | 1.62 (0.73-3.62) |
| Health literacy (sufficient) |  | 1.93 (0.92-4.03) | 1.53 (0.70-3.38) |
| Country of birth (Sweden) |  | 2.10 (0.85-5.18) | 2.08 (0.79-5.51) |
| Pharma use (daily) |  | 1.80 (0.79-4.10) | 1.80 (0.74-4.36) |
| Moral status of embryo |  |  | 7.86 (3.43-18.02) |

*OR: odds ratio, CI: confidence interval. Model 1 included religious views. Model 1 included religious views, age, sex, education, health literacy, country of birth, and use of pharmaceuticals. Model 2 included religious views, age, sex, education, health literacy, country of birth, use of pharmaceuticals, and perception of moral status of embryo.*

Table S2. The association of importance of religion in life with a positive attitude toward using embryos for treatment of II) of other diseases. Associations are presented using odds ratio (OR) and 95% confidence intervals (CI), and with respondents finding religion to be of fairly high or very high importance, as the reference groups (ref).

|  | Crude model  OR (95% CI) | Model 1  OR (95% CI) | Model 2  OR (95% CI) |
| --- | --- | --- | --- |
| Importance of religion in life (very little, fairly little, neither little nor a lot) | 6.49 (3.31-12.73) | 5.41 (2.68-10.94) | 3.47 (1.56-7.71) |
| Age |  | 0.98 (0.96-1.00) | 0.99 (0.97-1.02) |
| Sex (male) |  | 1.56 (0.83-2.96) | 1.65 (0.83-3.30) |
| Education (university) |  | 1.29 (0.68-2.46) | 1.55 (0.77-3.13) |
| Health literacy (sufficient) |  | 1.76 (0.93-3.33) | 1.47 (0.73-2.96) |
| Country of birth (Sweden) |  | 2.11 (0.95-4.70) | 2.11 (0.87-5.10) |
| Pharma use (daily) |  | 1.36 (0.68-2.73) | 1.37 (0.64-2.94) |
| Moral status of embryo |  |  | 10.38 (4.96-21.76) |

*OR: odds ratio, CI: confidence interval. Model 1 included religious views. Model 1 included religious views, age, sex, education, health literacy, country of birth, and use of pharmaceuticals. Model 2 included religious views, age, sex, education, health literacy, country of birth, use of pharmaceuticals, and perception of moral status of embryo.*

Table S3. The association of importance of religion in life with a positive attitude toward using embryos for treatment of III) of diseases, although iPS cells are as efficient. Associations are presented using odds ratio (OR) and 95% confidence intervals (CI), and with respondents finding religion to be of fairly high or very high importance, as the reference groups (ref).

|  | Crude model  OR (95% CI) | Model 1  OR (95% CI) | Model 2  OR (95% CI) |
| --- | --- | --- | --- |
| Importance of religion in life (very little, fairly little, neither little nor a lot) | 2.58 (1.42-4.69) | 2.29 (1.24-4.23) | 1.75 (0.92-3.34) |
| Age |  | 0.99 (0.97-1.00) | 0.99 (0.98-1.00) |
| Sex (male) |  | 1.67 (1.11-2.50) | 1.72 (1.14-2.60) |
| Education (university) |  | 1.02 (0.67-1.53) | 1.06 (0.70-1.61) |
| Health literacy (sufficient) |  | 1.11 (0.73-1.70) | 1.05 (0.68-1.62) |
| Country of birth (Sweden) |  | 1.08 (0.59-1.96) | 1.01 (0.55-1.88) |
| Pharma use (daily) |  | 1.19 (0.77-1.85) | 1.21 (0.77-1.90) |
| Moral status of embryo |  |  | 4.00 (2.07-7.64) |

*OR: odds ratio, CI: confidence interval. Model 1 included religious views. Model 1 included religious views, age, sex, education, health literacy, country of birth, and use of pharmaceuticals. Model 2 included religious views, age, sex, education, health literacy, country of birth, use of pharmaceuticals, and perception of moral status of embryo.*

Table S4. Correlations between religious views and moral views of the embryo

Cross-table. Pearson’s Chi^2^ test shows correlation p<0.001

|  | **Moral view of the embryo** | | | | |
| --- | --- | --- | --- | --- | --- |
| ***How great significance do religion has in your life?*** | Cell lump | In between cell lump and human | Closer to a human than a cell lump | Same as a human | Total |
| *very little* | 150 (66.4) | 56 (24.8) | 15 (6.6) | 5 (2.2) | 226  (100) |
| *pretty little* | 46 (54.8) | 35 (41.7) | 2 (2.4) | 1 (1.2) | 84  (100) |
| *neither little nor a lot* | 40 (54.8) | 20 (27.4) | 9 (12.3) | 4 (5.5) | 73  (100) |
| *pretty much* | 12 (37.5) | 14 (43.8) | 5 (15.6) | 1 (3.1) | 32  (100) |
| *very much* | 4 (21.1) | 3 (15.8) | 6 (31.6) | 6 (31.6) | 19  (100) |
| *Total* | 252 (58.1) | 128 (29.5) | 37 (8.5) | 17 (3.9) | 434  (100) |

Table S5. The association of importance of religion in life with moral status of embryo (perceiving the embryo as a cell lump or in between being just a lump of cells and being a human being). Associations are presented using odds ratio (OR) and 95% confidence intervals (CI), and with respondents finding religion to be of fairly high or very high importance, as the reference groups (ref).

|  | Crude model  OR (95% CI) | Model 1  OR (95% CI) |
| --- | --- | --- |
| Importance of religion in life (very little, fairly little, neither little nor a lot) | 5.24 (2.69–10.24) | 4.61 (2.27–9.36) |
| Age |  | 0.97 (0.95–0.99) |
| Sex (male) |  | 1.00 (0.54–1.84) |
| Education (university) |  | 0.74 (0.40–1.39) |
| Health literacy (sufficient) |  | 1.59 (0.85–2.97) |
| Country of birth (Sweden) |  | 1.49 (0.65–3.42) |
| Pharma use (daily) |  | 1.00 (0.51–1.96) |

*OR: odds ratio, CI: confidence interval. Model 1 included religious views. Model 2 included religious views, age, sex, education, health literacy, country of birth, and use of pharmaceuticals.*

Table S6: Distribution of Importance of religion and Attitude towards using embryos for treatment of Parkinson’s disease

|  | | It is acceptable to use embryos for treatment of Parkinson’s disease | | | | |
| --- | --- | --- | --- | --- | --- | --- |
|  |  | 1:Totally agree  n | 2  n | 3  n | 4  n | 5: Do not agree at all  n |
| Importance of religion in life | very little | 171 | 45 | 7 | 0 | 3 |
|  | pretty little | 61 | 21 | 1 | 0 | 1 |
|  | neither little nor alot | 49 | 16 | 3 | 3 | 2 |
|  | pretty much | 21 | 5 | 3 | 1 | 2 |
|  | very much | 4 | 2 | 5 | 2 | 6 |
|  | Total | 306 | 89 | 19 | 6 | 14 |

Table S7: Distribution of Importance of religion and Attitude towards using embryos for treatment of other diseases

|  | | It is acceptable to use embryos for treatment of other diseases | | | | |
| --- | --- | --- | --- | --- | --- | --- |
|  |  | 1:Totally agree  n | 2  n | 3  n | 4  n | 5: Do not agree at all  n |
| Importance of religion in life | very little | 162 | 46 | 12 | 3 | 3 |
|  | pretty little | 58 | 22 | 2 | 2 | 0 |
|  | neither little nor alot | 42 | 21 | 4 | 3 | 3 |
|  | pretty much | 17 | 9 | 3 | 1 | 2 |
|  | very much | 4 | 2 | 4 | 4 | 5 |
|  | Total | 283 | 100 | 25 | 13 | 13 |

Table S8: Distribution of Importance of religion and Attitude towards using embryos for treatment of other diseases although iPS cells are as efficient

|  | | It is acceptable to use embryos for treatment of other diseases although iPS cells are as efficient | | | | |
| --- | --- | --- | --- | --- | --- | --- |
|  |  | 1:Totally agree  n | 2  n | 3  n | 4  n | 5: Do not agree at all  n |
| Importance of religion in life | very little | 106 | 49 | 50 | 13 | 8 |
|  | pretty little | 30 | 23 | 18 | 8 | 5 |
|  | neither little nor alot | 26 | 13 | 21 | 6 | 7 |
|  | pretty much | 10 | 6 | 12 | 1 | 3 |
|  | very much | 3 | 2 | 1 | 0 | 13 |
|  | Total | 175 | 93 | 102 | 28 | 36 |
